# Development of a biosensor platform based on ITO sheets modified with 3-glycidoxypropyltrimethoxysilane for early detection of TRAP1

**DOI:** 10.3906/kim-1909-53

**Published:** 2020-04-01

**Authors:** Berfin VURAL, Meltem ÇALIŞKAN, Mustafa Kemal SEZGİNTÜRK

**Affiliations:** 1 Department of Bioengineering, Faculty of Engineering, Çanakkale Onsekiz Mart University, Çanakkale Turkey

**Keywords:** 3-GOPS, ITO-PET, TRAP1, immunosensor, biosensor

## Abstract

The aim of this research was to design an electrochemical immunosensor for determination of tumour necrosis factor receptor-associated protein-1(TRAP1) antigen, a heat shock protein linked to tumour necrosis factor. The indium-tin oxide covered polyethylene terephthalate (ITO-PET) electrode surface was cleaned and was prepared for the introduction of hydroxyl groups on its surface by using NH4 OH/H2 O2 /H2 O. As a silanization agent for covalent attachment of anti-TRAP1 on the surface of the ITO working electrode, 3-glycidoxypropyltrimethoxysilane (3-GOPS) was used. Cyclic voltammetry (CV) and electrochemical impedance spectroscopy (EIS) were used to characterize the immobilization steps. A variety of parameters, 3-GOPS and anti-TRAP1 concentrations, and anti-TRAP1 and TRAP1 incubation durations were optimized. After determining the optimum conditions, characterization studies such as repeatability, reproducibility, regeneration, square wave voltammetry, and single frequency impedance were performed. The electrochemical immunosensor has presented an extremely wide determination range for TRAP1 from 0.1 pg/mL to 100 pg/mL.

## 1. Introduction

Tumour necrosis factor receptor-associated protein-1 (TRAP1) is a protein linked to the tumour necrosis factor receptor [1]. Tumour necrosis factor is a communication protein playing a role in destruction of cancer cells. It is a glycoprotein with 185 amino acids and is coded on the 7th chromosome. The coded genes are found in the major histocompatibility complex (MHC). MHC provides information about autoimmune diseases. The TRAP1 gene is found on the 16p13 chromosome. Genome-wide association studies for systemic lupus erythematosus showed susceptibility genes in the 16p13 region [2]. This shows that TRAP1 is associated with autoimmune diseases, which involve situations where the immune system attacks normal tissue in the body because of an error. TRAP1 expression levels are also linked to tumour progression so it is a mitochondria chaperone protein stated to be a target for cancer treatment [3]. In the context of cancer cells, overexpression of TRAP1 and silencing were shown to cause sudden growth inhibition and apoptosis [4].

Diagnosis of the diseases is based on clinical symptoms of features and laboratory tests. In this context, biomarkers are a guide for the development of biosensors and are materials used to aid in identifying diseases, reflecting disease activities, predicting future symptoms, and directing pharmaceutical treatments. Moreover, detection of special and sensitive biomarkers for every autoimmune disease is an unmet need, which may assist in delaying initiation of symptoms with early intervention, reducing mortality rates and limiting organ injury, and especially for early diagnosis and prediction of flare-ups [5]. In line with this requirement, development of biosensors has vital importance for early identification of disease status and health disorders, surveillance of human health, and maintaining a healthy life. Biosensors identify target molecules and measure within a certain detection interval in a biological sample and are analytical measurement devices transforming this information into a meaningful electrical signal. In this study, polyethylene terephthalate sheet covered by ITO were used as a flexible and disposable working electrode material in order to develop a biosensor for early diagnosis of TRAP1. ITO is a promising material which has been widely used especially in electrochemical biosensing applications because of its unique properties such as low surface electrical resistance, electrochemical and physical stability, and easy to chemically modify. Moreover, their long shelf-life, extremely low cost than other conventional electrodes such as glassy carbon, gold, or screen printed electrodes, and disposable features make ITO covered electrodes more feasible for the construction of biosensors [6–16]. In the present study, 3-GOPS was effectively used for the covalent immobilization of anti-TRAP1 onto the ITO-PET surface. The immobilization steps were electrochemically monitored by CV and EIS methods. Optimization of the fabrication parameters and the characterization of the biosensor were also carried out in depth. Finally, the biosensor was applied to the real human serum samples for the determination of TRAP1 levels.

## 2. Materials and methods

### 2.1. Materials

All chemicals, anti-TRAP1 antibody, TRAP1 antigen, BSA protein, and ITO-PET films (surface permeability: 550 nm (>79%), resistance: 60 Ω/square) were purchased from Sigma Aldrich (USA). All proteins were prepared with phosphate buffer (pH 7.0) and stored at –20 °C.

### 2.2. Electrochemical experiments

All electrochemical measurements were performed using a computer-interface Compactstat with an integrated impedance analyser (Ivium Technologies, Eindhoven, The Netherlands). A PURELAB flex 3 & 4 Ultra-pure Water Treatment System (ELGA LC 134 model) was used in the preparation of ultrapure water for all solutions. In this study, a triple electrode system with reference electrode, counter electrode, and working electrode was used. As the reference electrode, Ag/AgCl (saturated with KCl) was used, ITO-coated PET film was used as the working electrode, and a platinum wire was used as a counter electrode. The reference electrode and counter electrode were purchased from BASi (West Lafayette, IN, USA).

**Figure 1:**
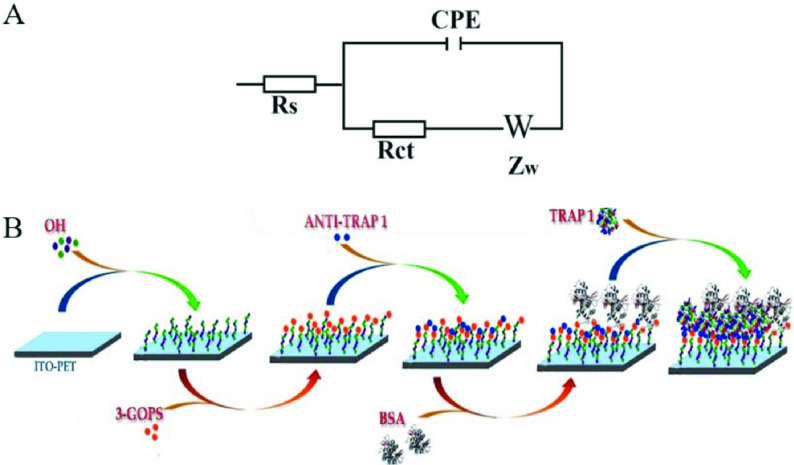
(A) Equivalent circuit model used for electrochemical impedance extraction. (B) Illustration of the immobilization steps for the TRAP1 immunosensor on the ITO-PET surface.

The performance of the immobilization steps and TRAP1 analysis were monitored by different electrochemical techniques such as EIS, CV, and SWV. For all these methods K3 [Fe(CN)6 ]/K4 [Fe(CN)6 ] solution containing 0.1 M KCl were used as an electrochemical redox probe solution. Unless otherwise stated, EIS experiments were carried out between the frequency range of 100 kHz (initial frequency) and 0.05 Hz (final frequency) under the DC potential of 0.05 V. Cyclic voltammograms were collected from –1 V to 1 V (scan speed: 100 mV/s, step size: 10 mV). For the calculation of impedance values, a simple equivalent circuit model was used. This model included charge transfer resistance (Rct) , the constant phase element (CPE), electrolyte resistance (Rs) , and Warburg element (W) as can be seen in Figure 1A. By the help of the circuit model the impedance value was extracted and the impedance variations between the surfaces were calculated by the following equation:

ΔRct = Rct(anti−TRAP1/TRAP1) - Rct(anti−TRAP1/BSA) where Rct(anti−TRAP1/TRAP1) is the value of the charge transfer resistance after anti-TRAP1 was coupled to TRAP1, while Rct(anti−TRAP1/BSA) is the value of the impedance before the TRAP1 was applied to the biosensor surface. Impedance data extraction from the impedance spectra was carried out by the equation given above for all EIS experiments.

### 2.3. Immobilization steps of the biosensor

First, the bare ITO-PET sheet was cleaned in acetone, soap solution, and ultra-pure water, respectively. All these cleaning steps were performed in an ultrasonic bath for 10 min. Next the cleaned sheet was treated with the mixed solution of NH4 OH, H2 O2 , and ultra-pure water with a ratio of 1:1:5 (v/v/v) for 90 min. Hereby –OH groups were introduced to the surface of ITO-PET working electrode. After that the electrode was gently immersed into the ultra-pure water to remove the excess of the hydroxylation solution and then the surface was dried by argon gas stream. In the next step, the working electrode carrying the –OH groups was modified with 3-GOPS by overnight incubation (nearly 16 h) in 3-GOPS solution in dark and cold environment (+4 °C). Similarly, the electrode was gently immersed into the ultra-pure water to remove the excess of 3-GOPS solution and then it was dried by argon gas stream. In order to provide covalent attachment of anti-TRAP1 to the electrode surface which introduced active epoxy ends, the electrode was immersed into the anti-TRAP1 solution with a certain concentration for 45 min. After the anti-TRAP1 immobilization, the electrode was treated with BSA solution (for 1 h) in case some active epoxy ends did not interact with anti-TRAP1. By the help of this process, active epoxy ends on the surface were blocked. Finally, the electrodes were washed with ultra-pure water and were gently dried with argon gas and were ready to be used. Figure 1B shows the immobilization steps.

## 3. Results and discussion

### 3.1. Fabrication steps of the biosensor

**Figure 2:**
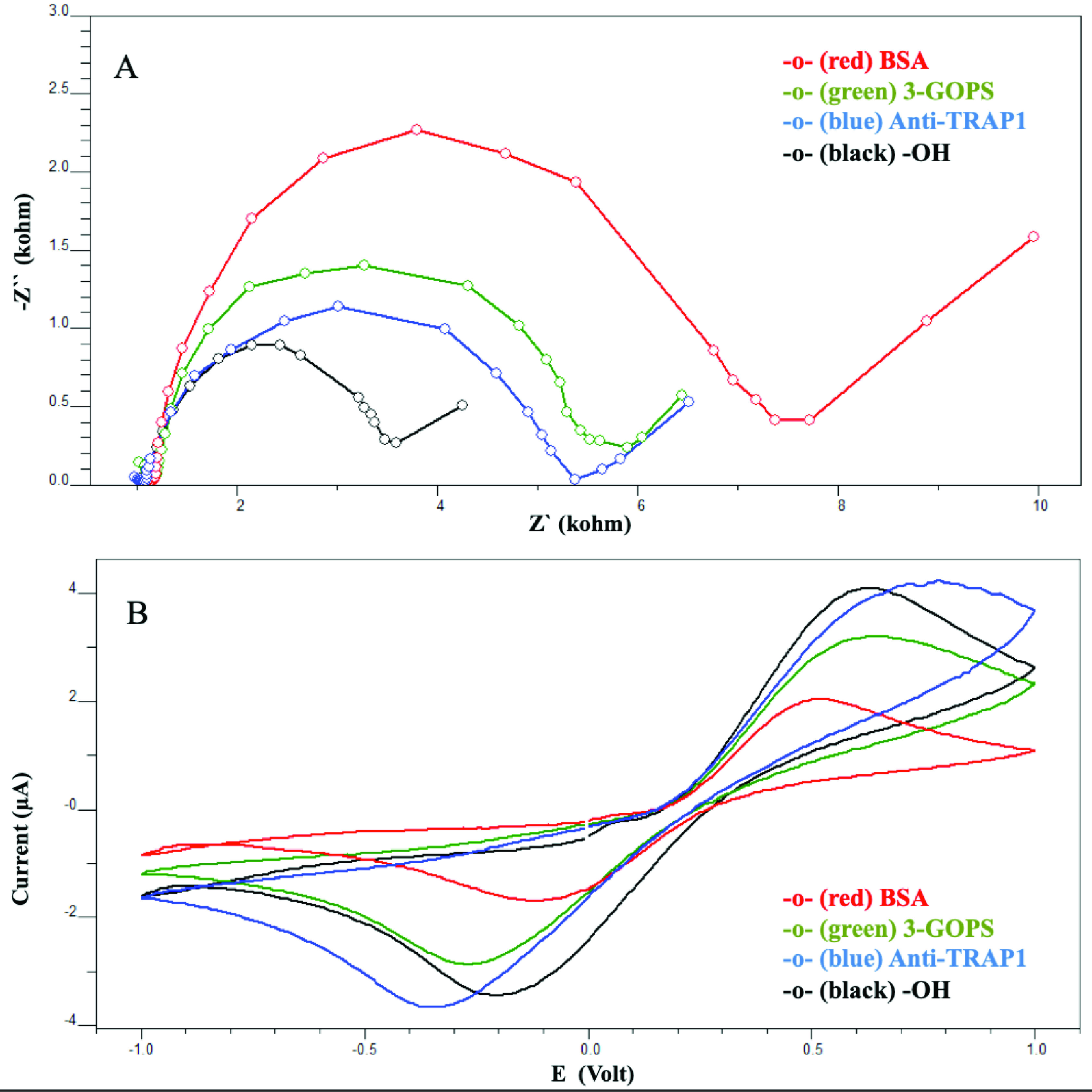
Immobilization steps of the biosensor [(A) Electrochemical impedance spectra obtained for the fabrication steps. (B) Cyclic voltammograms obtained for the fabrication steps.]

The effect of the immobilization steps on the biosensor surface was monitored by the electrochemical methods of EIS and CV. The results are given in Figure 2. As can be seen from the impedance spectra (Figure 2A), 3-GOPS modification caused an increase in the impedance value. This effect should be attributed to the unpaired electrons on the oxygen atom of the reactive epoxy ends. These electrons probably electrostatically repulsed the negatively charged redox probe. Consequently, the charge transfer resistance was increased. This effect has also been observed and reported previously [17,18]. After anti-TRAP1 immobilization the impedance value has slightly decreased. Even though this result seems a little bit interesting, it was also an expectable outcome. Because the diffusion barrier effect on the redox probe of anti-TRAP1 was not higher than that of the reactive epoxy ends. Consequently, anti-TRAP1 proteins made the diffusion of the redox probe to the electrode surface easy. However, more proteins by BSA modification caused a considerable insulating layer and resulted in increase in impedance value significantly. Cyclic voltammetry experiments have corroborated the EIS results. Modification of the electrode surface by 3-GOPS resulted in a decrease in peak currents because of the same effect mentioned in EIS. Peak currents slightly higher than the previous ones have been obtained after anti-TRAP1 interaction. Finally, BSA treatment for the blocking the unbound active epoxy ends gave rise to a decrease in peak currents (Figure 2B). Eventually EIS and CV experiments revealed that anti-TRAP1 was successfully immobilized onto the ITO electrode surface.

### 3.2. Optimization steps for the biosensor

**Figure 3:**
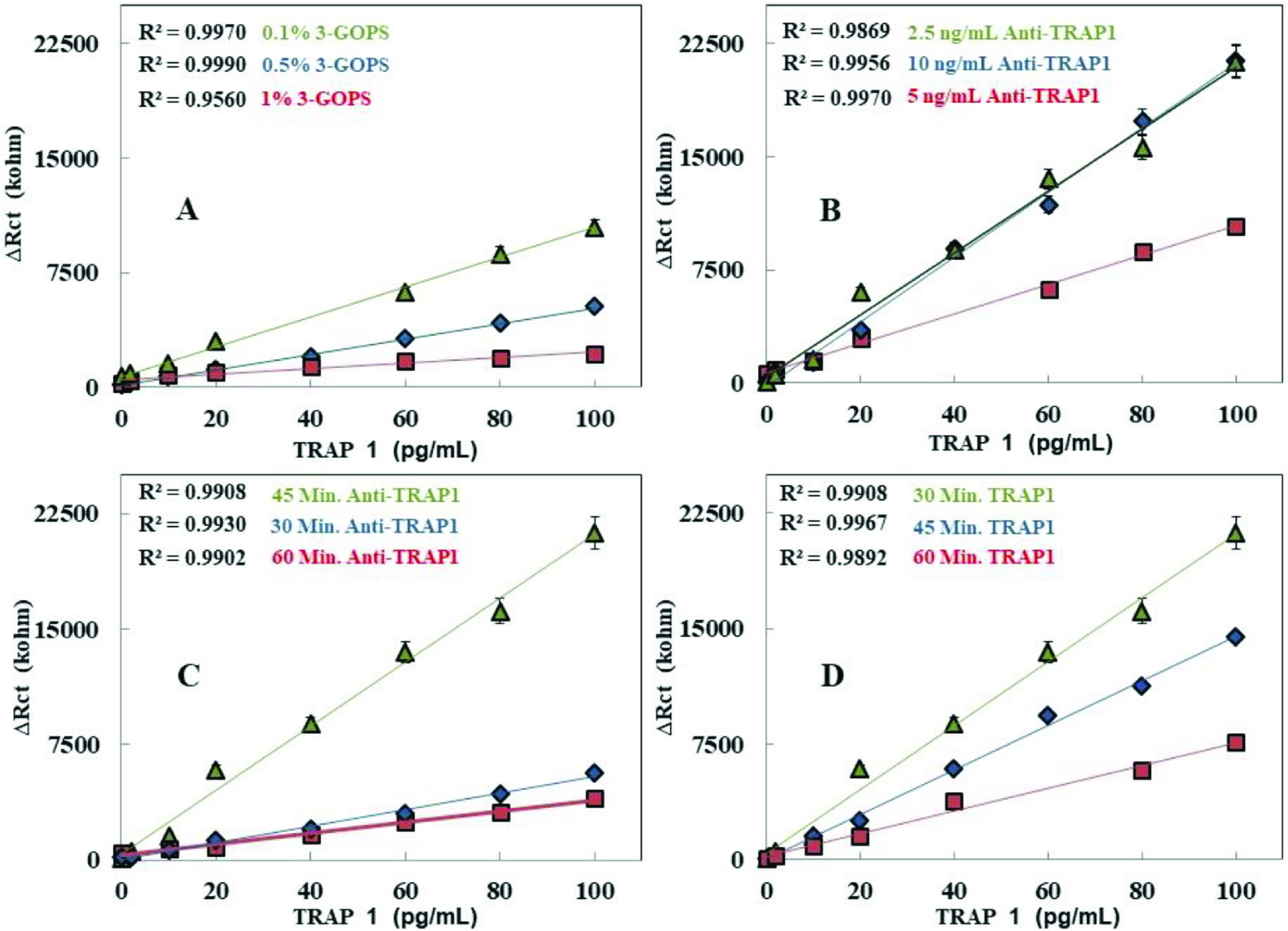
Optimization steps for the biosensor [(A) The effect of the 3-GOPS concentration on the biosensor performance. (B) The effect of anti-TRAP1 concentration on the biosensor performance. (C) The effect of anti-TRAP1 incubation duration on the biosensor. (D) The effect of TRAP1 incubation duration on the biosensor.]

Current optimization experiments for the biosensor fabrication were carried out. Optimization experiments were important because they were needed to obtain a biosensor with high electrochemical performance. Firstly, the effect of the concentration of 3-GOPS on the biosensor performance was investigated. For this purpose, different biosensors were fabricated by using 3-GOPS concentrations of 0.1%, 0.5%, and 1%, separately. The TRAP1 calibration plots obtained by them are compared in Figure 3A. The maximum Rct value was observed when 0.1% 3-GOPS concentration was used. Moreover, the biosensor prepared with 0.1% 3-GOPS concentration presented better and linear results for TRAP1 analysis. An increase in the concentration of 3-GOPS resulted in decrease in the Rct values obtained for TRAP1 standards. Actually, this was an expected result because the high concentrations of 3-GOPS probably formed a highly dense electrode surface which was negatively affected by the immobilization of the anti-TRAP1. According to these results, the most appropriate 3-GOPS concentration for the biosensor construction was selected as 0.1%.

The second optimization parameter was related to determine the optimum concentration of anti-TRAP1 immobilized onto the electrode surface. To determine how the response of the biosensor was affected by each anti-TRAP1 concentrations, 2.5 ng/mL, 5 ng/mL, and 10 ng/mL anti-TRAP1 concentrations were used to construct the biosensors. The calibration curves for TRAP1 obtained by the different biosensors, which were fabricated by using different anti-TRAP1 concentrations, are given in Figure 3B. When the different plots obtained for TRAP1 were evaluated, the most appropriate anti-TRAP1 concentration was seemed as 2.5 ng/mL. Additionally, though the signals for 2.5 ng/mL and 10 ng/mL anti-TRAP1 concentrations were very close to each other, it was decided to use the lower concentration with the aim of preventing waste of protein. As a result, the most appropriate anti-TRAP1 concentration for the biosensor was chosen as 2.5 ng/mL. The next optimization parameter was anti-TRAP1 incubation period. In this stage, the incubation durations of 30 min, 45 min, and 60 min were used to immobilize the chosen anti-TRAP1 concentration on the modified surface of the electrode. The calibration curves for different anti-TRAP1 incubation durations are given in Figure 3C. The longest incubation duration (60 min) caused a reduction in charge transfer resistance. This is most probably due to surface damage. Damage may be caused by high antibody amounts at the surface. Moreover, shorter periods than 45 min were not sufficient for the effective immobilization of anti-TRAP1 onto the electrode surface. According to these results, the most appropriate anti-TRAP1 incubation duration for the biosensor fabrication was 30 min. Finally, as the most important parameter related to biosensor operating, TRAP1 incubation period was investigated by the interaction of the electrode surface with varying TRAP1 incubation periods. For this purpose, the experiments were carried out by using periods such as 30, 45, and 60 min. The calibration plots for TRAP1 can be seen in Fig 3D. When the calibration curves are investigated, the appropriate period for the interaction between TRAP1 and anti-TRAP1 immobilized on the surface was 30 min. Because longer periods than 30 min for TRAP1 incubation gave rise to a dramatic decrease in charge transfer resistances. This result can be attributed to the negative effect of high dense of electrode surface. Besides, when TRAP1 was incubated for longer than 30 min, the dissociation of TRAP1 proteins from the electrode surface could probably happen.

### 3.3. Characterization of the biosensor performance

**Figure 4:**
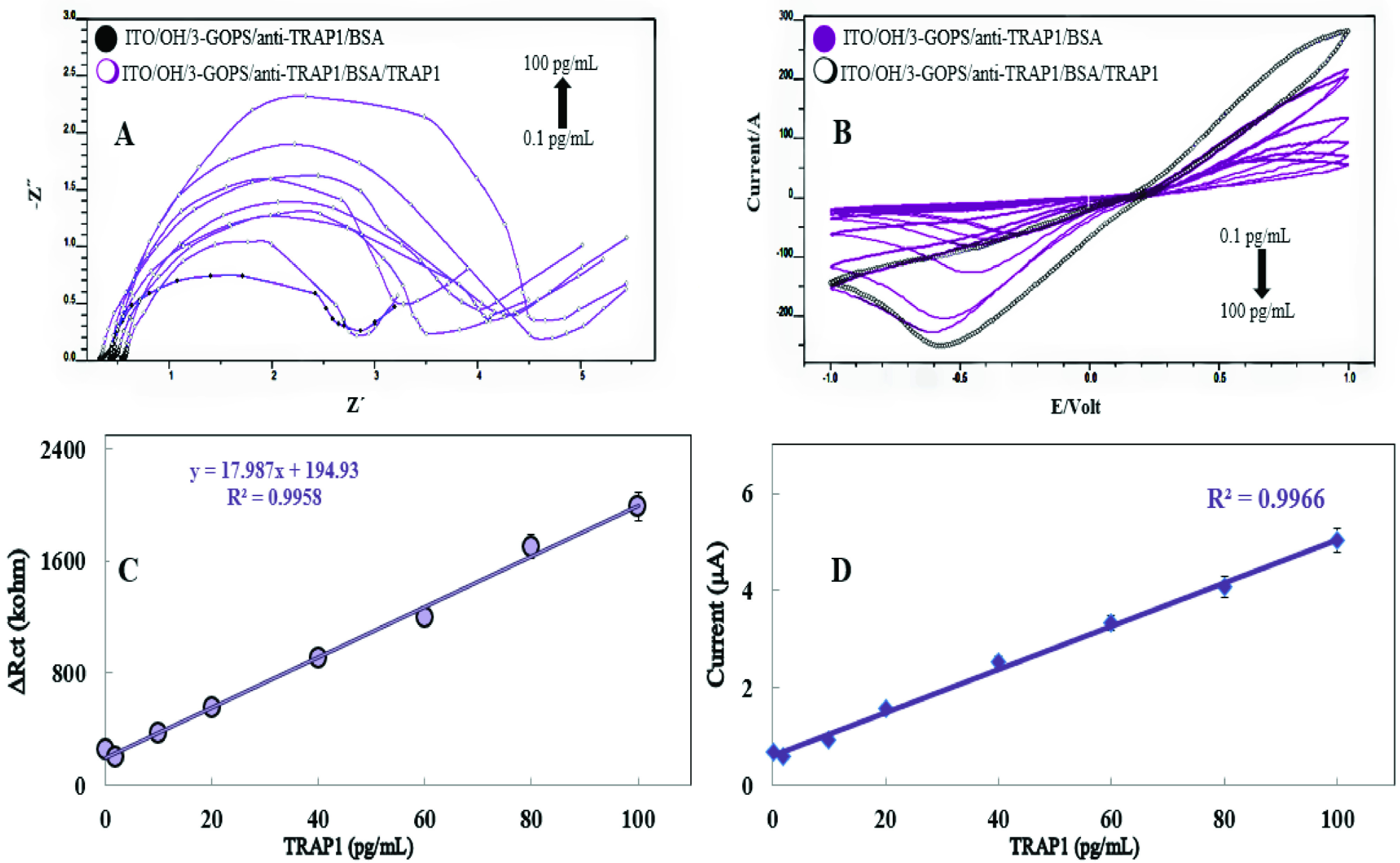
Characterization experiments of the biosensor [(A) EIS experiments for standard calibration plot of TRAP1. (B) CV experiments for standard calibration plot of TRAP1. (C) Linear calibration graph for TRAP1 obtained by EIS. (D) Linear calibration graph for TRAP1 obtained by SWV.]

After the optimization experiments a TRAP1 calibration graph was plotted at the optimum working conditions with the biosensor fabricated by optimum preparation combination. Figure 4A and 4B show the variations in the electrochemical impedance spectra and cyclic voltammograms obtained for the increased concentrations of TRAP1, respectively. As can be seen from the Figure 4A, an increase in TRAP1 concentration caused an increase in impedance as expected. Similarly, a decrease in peak currents by increasing concentrations of TRAP1 was observed in cyclic voltammograms (Figure 4B). In both cases these results can be attributed to the blocking effect of the additional protein layer on the diffusion of the redox probe to the electrode surface. A calibration graph drawn in the direction of the impedance spectra is shown in Figure 4C.

The biosensor represented linear calibration range between 0.1 pg/mL and 100 pg/mL of TRAP1. A 1000-fold linear range for TRAP1 analysis is a perfect analytical characteristic. Additionally, important analytical features, limit of detection (LOD) and limit of quantification (LOQ) were also calculated. A simple equation [k*SS/m], was used to calculate these values. In this equation, k is a constant which is accepted as 3 in LOD and as 10 in LOQ, SS (standard deviation), and m (slope of the calibration curve obtained by EIS). By the equation, LOD and LOQ were calculated as 0.217 pg/mL and 0.72 pg/mL, respectively. Another electrochemical method, square wave voltammetry was also applied to the biosensor to obtain a TRAP1 calibration graph. The working conditions and fabrication features were the same as the impedance experiments. The graph of observed peak current against increasing TRAP1 concentration was drawn. The TRAP1 calibration graph obtained by SWV is given in Figure 4D. As can be extracted from the figure, SWV could also be successfully used for the biosensor as an electrochemical method. Another important 2 characterization experiments were repeatability and reproducibility. These terms are often confused although, repeatability and reproducibility differ greatly from each other. Repeatability of a biosensor represents a ratio of agreement between the results of consecutive quantitative analysis of a same biological target carried out under the identical working conditions. On the other hand, reproducibility represents closeness of the agreement between the electrochemical signals obtained for the same target with the different biosensors which are fabricated by the same methodology. In order to evaluate the repeatability of the developed biosensor, 20 successive analyses of 40 pg/mL of TRAP1 standard solution were carried out by the 20 disposable biosensor. To calculate the found TRAP1 concentration by the biosensors the equation given in Fig.4C was utilized. In the light of these data, mean TRAP1 concentration found by the biosensor, the standard deviation, and the variation coefficient were calculated as 40 pg/mL, ±1.3 pg/mL, and 1.7%, respectively. These results showed that the disposable biosensor based on ITO had a perfect repeatability even at relatively high TRAP1 concentrations and made sensitive TRAP1 analysis possible with a high repeatability.

**Figure 5:**
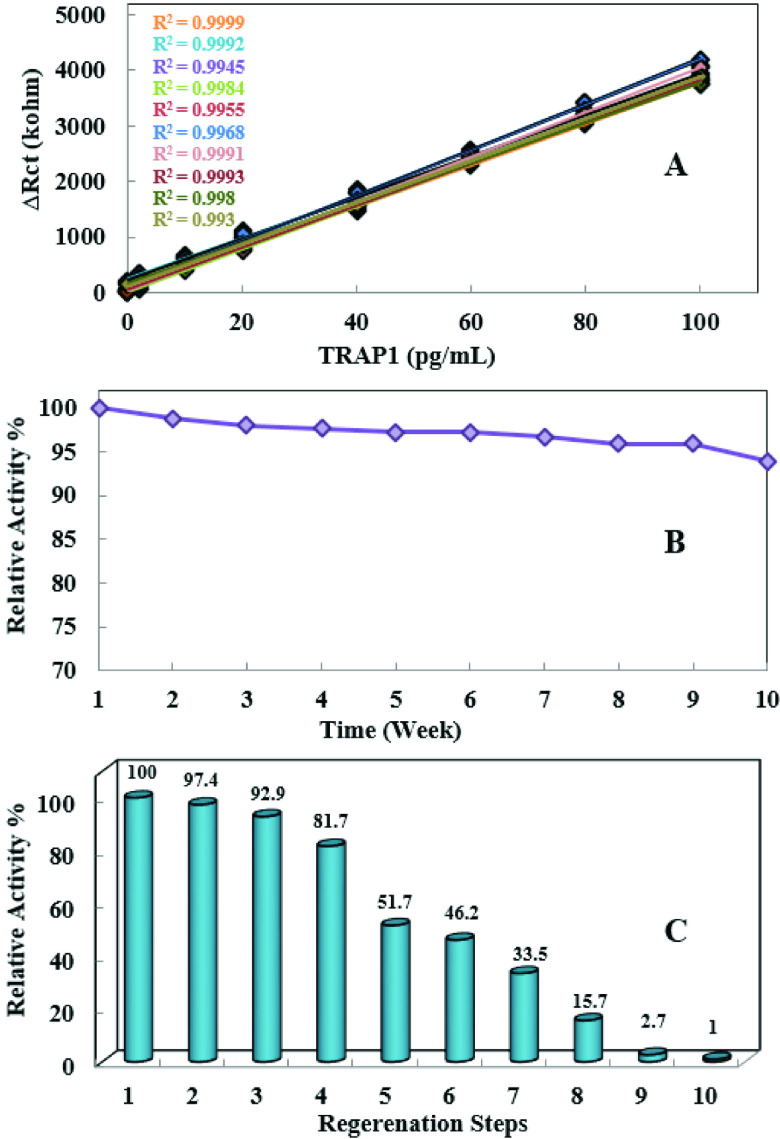
(A) Reproducibility of the biosensor. (B) Storage stability of the biosensor. (C) Regeneration experiment results for the biosensor.

For evaluation of the reproducibility of the biosensor, 10 calibration plots for TRAP1 were prepared. For this purpose, 80 pieces of disposable biosensors were fabricated with the same characteristics. 10 calibration graphs can be found in Figure 5.A. As can be seen from the figure the impedance signals obtained for the same concentrations were very close to each other which strongly proved the extremely high reproducibility of the designed disposable biosensor. Moreover, R2 values of each linear calibration plot were also perfect.

The storage life of the TRAP1 immunosensor was also investigated. ITO-PET electrodes were stored at 4 °C for 10 weeks. Every week, for making the impedance measurements, an electrode was performed in the analysis of 40 pg/mL TRAP1 standard solution. In order to calculate the found TRAP1 concentration the equation given in Figure 4C was used. The disposable TRAP1 biosensor based on ITO demonstrated relatively long shelf-life of at least 10 weeks and it maintained its initial activity above 90% after being kept at 4 °C for the period of storage. In other words, the biosensor only lost 6.1% of its initial activity at the end of the 10 weeks’ storage (Figure 5B). In can be concluded that due to the low activity loss, the developed biosensor appears to have a long shelf-life.

**Figure 6:**
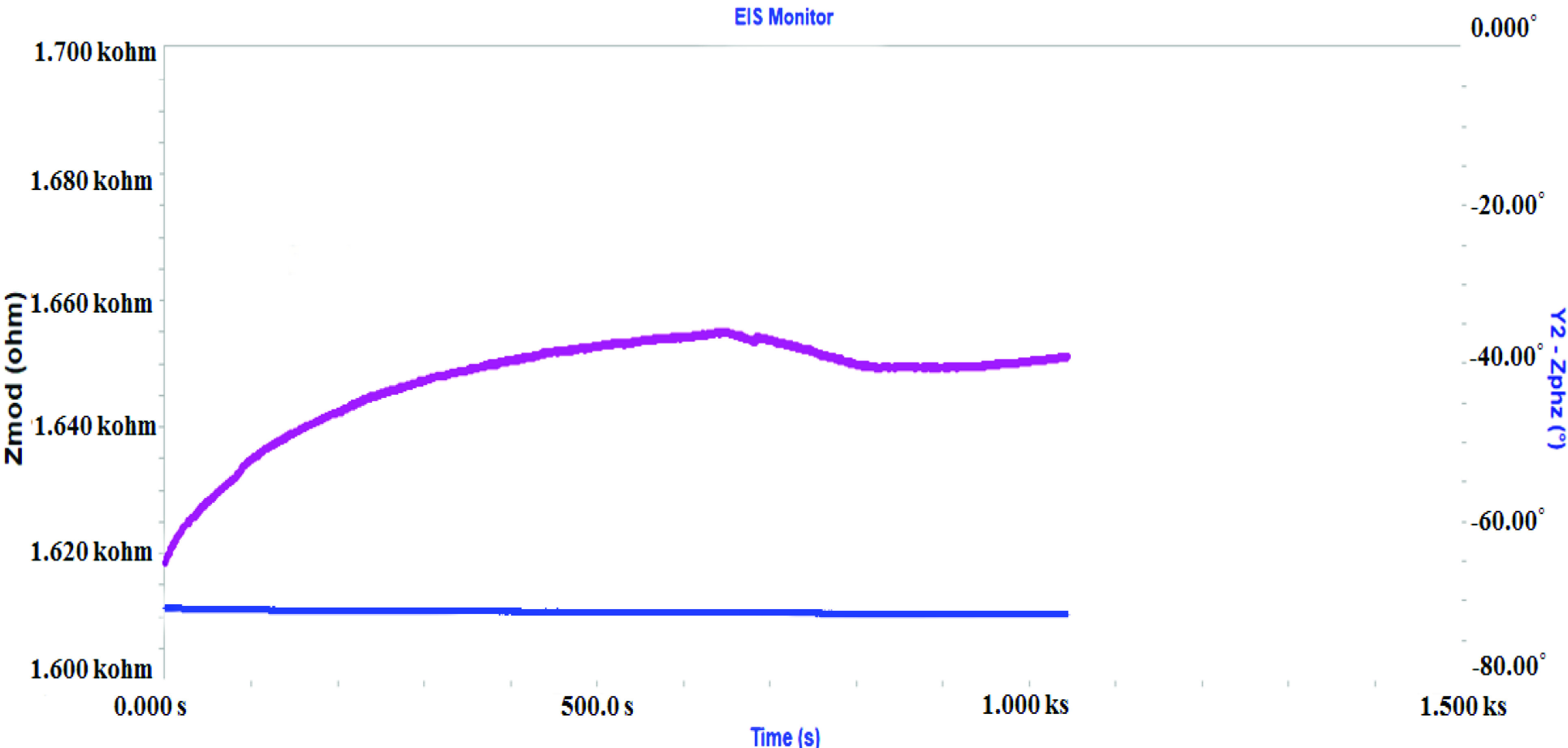
Single frequency impedance experiment for TRAP1 biosensor.

Although the developed biosensor had a single-use concept, for the multiple use, the regeneration possibility of the biosensor was also evaluated. As known a typically antibody-antigen bound is established by noncovalent interactions such as hydrogen bound, ionic or hydrophobic interactions, etc. As well known, these kinds of weak interactions are strictly depended on pH value. Consequently, to dissociate TRAP1 from the anti-TRAP1 immobilized onto the surface 1% HCl solution which was terminated or disrupted the interaction between anti-TRAP1 and TRAP1 was used. The results of these experiments are given in Figure 5C. The observations may be interpreted as the ITO-PET electrode having capacity for repeated use, in spite of being disposable. Thus, it may possible to use the ITO-PET electrodes one more time. Single frequency impedance experiments were also carried for the biosensor to show interaction between TRAP1 and anti-TRAP1, simultaneously. SFI is an impedance-based technique which is used for the monitoring changes in electrochemical impedance versus time at a constant frequency. Accordingly, SFI does not give a spectrum unlike standard impedance measurement. For the biosensor, SFI measurements were performed in pH 7 phosphate buffer solution containing TRAP1 (40 pg/mL). The single alternating current frequency applied to the biosensor was 80 Hz, which was determined by the related Bode plots. Figure 6 shows the SFI experiment.

As seen, an increase in impedance value was observed in time. This increase was obviously attributed to the interaction between TRAP1 and anti-TRAP1 and SFI technique proved that there was a bonding between antigen and antibody.

### 3.4. Real sample analysis

Finally, the immunosensor was applied to the analysis of TRAP1 content of real human serum samples. For this aim, standard addition method was used and real samples were prepared as contained standard concentrations of TRAP1. Standard addition method is especially important when the sample matrix affects the analytical characteristics of the biosensor. Real human serum samples may be evaluated as a complex matrix that could affect the analytical sensitivity of the biosensor. In the experiments, 5 different real human serum samples were analysed.

Before addition of TRAP1 to the serums, they were diluted 1000 times with ultra-pure water. Then, serum samples were spiked with 2 different TRAP1 concentrations (2 ng/mL and 10 ng/mL). As can be seen, the biosensor sensitively detected TRAP1 in human serum (Table).

**Table:** Application of the developed biosensor to real human serum.

Serum samples	TRAP1 measured (pg/mL)	Spiked TRAP1 (pg/mL)	TRAP1 total measured (pg/mL, n = 3)	RSD (%)	Recovery (%)
1	29.41	2 10	30.92/31.47/31.92 39.09/39.48/39.53	0.06 0.12	100.06 99.88
2	28.69	2 10	30.53/30.80/29.80 38.36/38.70/38.64	0.31 0.12	99.69 99.88
3	13.67	2 10	16.07/16.02/15.35 22.63/24.19/23.74	0.14 0.15	100.14 99.85
4	25.19	2 10	26.75/26.86/27.13 35.14/35.81/35.31	0.28 0.23	99.72 100.23
5	25.77	2 10	27.97/27.08/27.30 35.36/35.59/35.81	0.32 0.19	99.68 99.81

## 4. Conclusion

In this study, a new immobilization platform for the biosensing applications was reported. Disposable ITOPET based working electrodes provided a highly sensitive, stable, and cost-effective material for fabrication of the biosensor. As a silanization agent 3-GOPS was used and it was well accommodated with the surface of hydroxylated ITO-PET. Because 3-GOPS had active epoxy ends there was no required additional activation process or crosslinking agent such as glutaraldehyde. Optimum working and fabrication conditions were determined in detailed. The biosensor represented an extremely wide linear calibration ranging from 0.1 pg/mL to 100 pg/mL with LOD of 0.217 pg/mL and LOQ of 0.7248 pg/mL. Repeatability and reproducibility of the biosensor were also perfect. Storage stability of the biosensor should be improved. Because the developed system was a disposable, regeneration degree of the surface could be acceptable. Furthermore, the real sample analysis showed that the new biosensor was successfully applied to the TRAP1 determination of the real human serum samples. As a conclusion, for TRAP1 analysis, disposable, reproducible, inexpensive, and highly sensitive biosensor was successfully fabricated.
